# Impact of an integral accompaniment program on undergraduate students’ self-perception of transversal competence development: a quasi-experimental study

**DOI:** 10.3389/fpsyg.2025.1653779

**Published:** 2026-01-16

**Authors:** Paula Crespí, Santiago Álvarez-Montero, Amalia Faná del Valle Villar, Cruz Santos, Raúl Castañeda-Vozmediano

**Affiliations:** 1 Accompaniment Institute, Universidad Francisco de Vitoria, Madrid, Spain; 2 Faculty of Medicine, Universidad Francisco de Vitoria, Madrid, Spain; 3 Faculty of Health Sciences, Universidad Francisco de Vitoria, Madrid, Spain; 4 Faculty of Experimental Sciences, Universidad Francisco de Vitoria, Madrid, Spain

**Keywords:** integral accompaniment, humanization, health careers, transversal competences, soft skills development, subject, higher education, questionnaire

## Abstract

**Introduction:**

In the current healthcare context, accompaniment has emerged as a key practice for the humanization of care, distinguishing itself from other forms of support such as tutoring or coaching. This need for humanization requires the development of transversal competences, which have traditionally been marginally addressed in university education.

**Method:**

A quantitative quasi-experimental study with a pre-test/post-test design and no control group was designed to evaluate the impact of an integral accompaniment program on first-year students from various faculties, including Medicine and Health Sciences. The sample included 1,211 students from the academic years 2020–21, 2021–22, and 2022–23. The validated Basic Generic Competences Questionnaire (BGCQ) was used to measure self-perceived development of intra- and interpersonal competences.

**Results:**

The results showed significant improvements in both competence dimensions following the intervention (*p* < 0.001), with more pronounced gains in the intrapersonal dimension (Cohen’s *d* = 0.349) than in the interpersonal dimension (Cohen’s *d* = 0.146). Improvements were observed in subdimensions such as deep look, personal development, and effective communication, along with their associated competences. No significant improvements were found in the *teamwork* subdimension.

**Discussion:**

The integral accompaniment program proved effective in enhancing the self-perception of key competences related to the humanization of healthcare. Although the results are promising, particularly among students from the Faculties of Medicine and Health Sciences, further studies are needed to evaluate the sustainability and long-term impact of such interventions on professional training.

## 1 Introduction

According to the Cambridge Dictionary (n.d.), to humanize is “to make something less unpleasant and more suitable for people”. A more complex and complementary definition might be: “to make something more pleasant and responsive to their needs and desires in order to achieve the best possible life.” This definition implies placing something at the service of human beings to facilitate their personal fulfilment (Bermejo Higuera, 2014). Martín Urrialde (2021) defines humanization as “the approach of the healthcare system to the patient from a human perspective, enhancing empathy and integral care, as the patient is the raison d’être of the healthcare system and its professionals” (p. 309). The author also emphasizes the importance of treating the patient as a person, not reducing them merely to an illness or pathology. Busch et al. (2019) identify key elements of humanized health care as “respect for the patient’s dignity, uniqueness, individuality, and humanity, as well as adequate working conditions and sufficient human and material resources” (p. 461). Thus, to humanize means recognizing and protecting the intrinsic dignity of every person (Beesley, 2021) and humanizing healthcare systems, therefore, involves working toward a safer and more person-centered healthcare system, encompassing patients, families, caregivers, and healthcare professionals. A humanized healthcare system is one in which the primary purpose is to serve people, designed and structured to provide appropriate care (Zhao et al., 2016). Meeting this challenge requires understanding that human beings are not merely bodies in need of repair. From both psychological and philosophical perspectives, that is, namely philosophical anthropology, people are the sum of their feelings, beliefs, values, aspirations, preferences, and relationships (Beorlegui, 2017; Pose, 2022). One of the most important factors in the humanization of healthcare systems is precisely the nature of interpersonal relationships (Reyes-Téllez et al., 2024). There is growing scientific evidence of the importance of interpersonal relationships in human health and quality of life (Szalados, 2021). Specifically, the *Harvard Study of Adult Development*, one of the most extensive and comprehensive longitudinal studies ever conducted, aims to identify the factors which contribute to a long, healthy, and fulfilling life (Harvard Second Generation Study, n. d.). Among its findings, the quality of personal relationships stands out as the strongest predictor (Mineo, 2017). In recent decades, numerous scientific studies have focused on the importance of accompaniment within healthcare settings. A search for the term *“accompaniment”* on PubMed yields 1,428 results, seeing exponential growth in the last two decades. This number rises to 246,609 results when the search strategy is expanded to include various forms of accompaniment. Key issues related to the concept of accompaniment include its content, significance, evidence of its benefits for personal and professional well-being, and its specific impact across different sectors. Similarly, the relevance of accompaniment in university contexts has been highlighted by recent comparative analyses of programs implemented in Spanish institutions (García-Cardo et al., 2023).

The concept of humanization in healthcare has been the subject of rigorous academic analysis, highlighting its complexity and multidimensional nature. Rather than assuming a self-evident meaning, recent studies have applied systematic concept analysis methods to clarify its attributes and implications. For instance, Henao-Castaño et al. (2021), using Rodgers’ evolutionary method, define humanization as an integral approach that encompasses respect for dignity, person-centered care, and empathetic communication. Similarly, Taghinezhad et al. (2022) identify nine attributes of humanistic care in nursing, including presence, constructive interaction, and scientific competence. Ghanbari-Afra et al. (2022), following Walker and Avant’s method, reinforce these findings by emphasizing ethical commitment and relational quality as core elements. These studies share the following attributes of humanized care: respect for the dignity of the person and their rights, presence and person-centered care, empathetic and constructive communication, and scientific excellence. These analyses converge on the idea that humanization is not merely descriptive but normative, requiring deliberate strategies to integrate these attributes into professional practice and education.

In healthcare, interpersonal relationships can take different forms, each shaping how professionals and patients interact and collaborate. One example is the deliberative clinical relationship, in which professionals and patients work together to find the best possible course of action in the face of a health problem (Gracia and Lázaro, 2004; Seoane, 2016). Mentoring and tutoring focus on guiding less experienced professionals through advice and technical training (Álvarez-Montero et al., 2023), while coaching aims to enhance performance or achieve specific goals, whether for patients or healthcare staff (Todorova, 2022). Counseling is another approach, where a professional advises, guides, or counsels people with health problems (Hack et al., 2023). Although these approaches contribute to professional development and patient care, they tend to prioritize problem-solving or skill acquisition rather than the holistic or integral growth of the person. While these models offer valuable contributions to healthcare practice, they often operate within specific objectives—such as solving problems, improving performance, or transferring technical knowledge—without necessarily embracing a broader vision of personal development.

While these models offer valuable contributions to healthcare practice, they often operate within specific objectives—such as solving problems, improving performance, transferring technical knowledge, or developing both technical and transversal competencies—without necessarily embracing a broader vision of integral development of the person. This opens the door to a more comprehensive or integral perspective: accompaniment. Unlike approaches that focus primarily on tasks or outcomes, accompaniment proposes a relational paradigm oriented toward the integral growth of the person, encompassing both the accompanied and the accompanier. Accompaniment can be understood as a style of relationship or “a relational paradigm that responds to the growth of the person, both of the accompanied and of the accompanier” (González Iglesias, 2023, p. 21). As defined by Crespí and López (2023), it is “an intentional pedagogical action that aims to help and support people in their effort to know themselves and take decisions that favor their personal growth and development, with the necessary support in its implementation” (p. 2). A recent scoping review of academic publications reinforces this perspective, describing educational accompaniment as “an intentional educational action through which one person enlightens and supports another in their integral development, facilitating their autonomy, maturity, and realization of a fulfilling life” (López González et al., 2025, p. 1). Accompaniment is a relational process, that recognizes the unique value of each person, grounded in respect for dignity and freedom, and oriented toward integral development of the person (González-Iglesias and de la Calle Maldonado, 2020; Viñado Oteo and Miró López, 2022). However, the research of López González et al. (2025) indicates that, other relational practices—such as tutoring, mentoring, pedagogical guidance, and informal encounters—can be understood as forms or types of accompaniment when approached from a holistic perspective that seek the integral growth of the person being accompanied. This conceptual distinction justifies the focus of this study on accompaniment as a transformative strategy for humanizing healthcare education.

It is logical to think that the healthcare system needs to be humanized if there are indications the system itself harms those within it (professionals, patients, colleagues) or data suggesting that valuable expectations are not being met, expectations that promote the integral development of individuals. For example, a systematic review on global burnout rates among healthcare professionals found that more than one-third of public healthcare workers suffer from burnout (Nagarajan et al., 2024). Several studies have found that a high percentage of patients and health professionals considered the system to be lacking compassion (Francis, 2013; Henao-Castaño et al., 2021; Lown et al., 2011, 2017). Thus, “there is a lack of trust from patients toward the health care system, as well as feelings of stress, exhaustion, and fatigue among professionals” (Reyes-Téllez et al., 2024, p. 1). When a high percentage of patients and professionals believe that health systems are not compassionate, the only conclusion is that the quality of human relationships needs to be improved.

In the field of healthcare, accompaniment is essential not only in terms of care but also in aspects related to research. In this case, it involves addressing the training of researchers and the conduct of research in an integral manner. Furthermore, it means accompanying participants in clinical trials. This involves being by their side, keeping them informed, preventing them from feeling like mere objects of study, and ensuring they perceive themselves as people who are giving of themselves to foster scientific advances that benefit society, safeguarding their well-being and valuing the generous act they perform (Pietilä et al., 2019; Resnik, 2024).

The complexity of the problem is enormous and has been addressed by institutions such as the Picker Group, which conducts research to understand, evaluate and improve the experiences and needs of people in healthcare, ensuring that everyone involved in the healthcare system takes them into account. It analyzes the various needs of patients and professionals based on their experiences: from waiting lists, patients’ need to participate in health decision-making, to the well-being of professionals, a key factor in providing integral quality care (Picker Institute Europe, n.d.). Additionally, there is evidence that humanized medicine is cost-effective (Trzeciak and Mazzarelli, 2019). Humanizing healthcare is a complex process that includes addressing healthcare policies, culture, improving human relationships, professional training (Bermejo Higuera, 2014). In this regard, it is essential to consider the process of professional socialization, understood as the ongoing and dynamic process through which individuals internalize the knowledge, skills, attitudes, norms, and values that shape their professional identity (Jawula Salisu et al., 2019; Moon and Chang, 2023). This process is not only shaped by formal education but also by the influence of professional socializing agents—such as clinical teachers, mentors, and preceptors—who act as role models and play a key role in teaching transversal competences through example and relational experience (Jawula Salisu et al., 2019; Moon and Chang, 2023).

This study aims to contribute to the humanization of healthcare through an integral university program that produces more person-centered professionals (patients, family, team members, colleagues, administrative staff, etc.). The integral development of university students requires education in competences, both technical and transversal. Soft skills or transversal competences are considered an essential aspect of an integral education (Casanova Romero et al., 2020; Crespí et al., 2025a, b; Ruiz-Morales et al., 2017; Tejeda Díaz, 2016). They are also considered key competences for training professionals who are agents of humanization (Condori Salluco and Choque Rojas, 2022; D’Ottavio, 2023; García-Salido et al., 2019; Henao-Castaño et al., 2021). The concept of competences refers to: “the dynamic set of knowledge (knowing), skills or abilities (knowing how to do), attitudes, values and universal principles (knowing how to be) that, internalized and embodied in our acts, behaviors or ways of doing, put us on the path to our own excellence, plenitude and happiness” (Crespí, 2018, p. 128). Specifically, the university program proposed in this study, aimed at educating a more humane professional, involves training in generic, transversal competences or soft skills. These competences are necessary for any discipline and are highly in demand in the professional field (Caggiano et al., 2020; Qizi, 2020; Tang, 2019; Tripathy, 2021). The Organization for Economic Co-operation and Development, in their project called The Future of Education and Skills 2030 (OECD, 2018), and the European Commission (2017) also recognize the importance of these competences. In this regard, the European Higher Education Area (EHEA) itself refers to the need to instill these competences (Martínez Clares and González Morga, 2018; Munuera Gómez and Navarro Asencio, 2015; Saravia Domínguez et al., 2024). However, in reality they are largely overlooked in the academic curricula of degree programs (Vera and Tejada, 2020; Reyes-Téllez et al., 2024). In fact, there is a noticeable lack of coherence between institutional declarations and the specific actions implemented to promote the development of transversal competences. The ability of universities to adequately train students in these competences demanded by the labor market has even been questioned (Igwe et al., 2022). Similarly, the training and development of these competences are not usually explicitly addressed in the various subjects within health sciences (Palés-Argullós and Nolla-Domenjó, 2016; Vargas and Zaldivar, 2023). Although evidence of their effectiveness is emerging (Donnon et al., 2013; Papadakis et al., 2005), work remains to be done regarding professionalism, compassion, communication, teamwork, and lifelong learning (Van der Vleuten, 2015).

Specifically, this study presents a curricular program in transversal competences integrated into the curriculum of students at Universidad Francisco de Vitoria (UFV), with a special focus on students of health sciences. The aim is that such training will prepare them to be agents of humanization within healthcare systems. Therefore, the goal is to graduate professionals who possess the necessary transversal competences, both intra- and interpersonal, to establish satisfactory relationships with their future colleagues, patients, and colleagues, and who also have a personal experience of accompaniment, as defined above. In this regard, the main objective of the research is to determine the self-perception of UFV students regarding their transversal, intra- and interpersonal competences after the intervention conducted over three academic years, through the subject Skills and Competences of the Person (SCP). The choice to address the issue of humanization through a university integral program (SPC) is based on the understanding that humanized care requires more than technical competences—it demands the development of transversal competences. Integral education programs, such as SPC, are designed to foster transversal competences. Empirical evidence supports this approach: Reyes-Téllez et al. (2024) identified that training in communication is essential to overcoming barriers to humanized nursing care, while Tutor et al. (2023) demonstrated that early assessment and development of transversal competences in medical education significantly improve students’ attitudes toward patient-centered care. These findings reinforce the relevance of integral programs in cultivating professionals capable of delivering compassionate, respectful, and humanized healthcare.

The research hypotheses are as follows (H):

*H1*: Students from the entire university, as well as from each individual faculty, significantly improve their self-perceived development of intra- and interpersonal transversal competences.

*H2*: There are no significant differences in the self-perceived development of intra- and interpersonal transversal competences among students from different groups, such as faculties and academic cohorts.

## 2 Methodology

### 2.1 Design of research and variables

The research project consisted of a quantitative quasi-experimental and longitudinal study (measuring variables before and after intervention) without a control group.

The principal independent variable is the moment of measurement (before and after intervention); and the dependent variables are the intrapersonal transversal competences of self-awareness, self-acceptance and self-management, search for meaning in life, orientation to excellence and proactivity; and the interpersonal competences of cooperative work, work environment management, orientation towards results, verbal communication, paraverbal and non-verbal communication and communication for encounter. Other independent variables are gender, age, faculty and cohort.

### 2.2 Population and sample

The target population of the study consists of first-year undergraduate students at the UFV from the 2020–21 cohort, with 8,734 students; the 2021–22 cohort, with 9,743 students; and the 2022–23 cohort, with 10,595 students, totaling 29,072 students.

To select the sample, the following criteria were used: (1) inclusion criteria: students enrolled in the SCP subject during the 2020–21, 2021–22, and 2022–23 academic years who voluntarily chose to participate in completing the self-assessment survey on the development of transversal competences; and (2) exclusion criteria: students not enrolled in the SCP subject during those academic years, as well as enrolled students who declined to participate or did not complete the subject.

This study includes a total of 1,211 students who completed the questionnaire both before and after the intervention. Of the 1,211 students: 664 (54.8%) belong to the 2022–23 cohort, 310 (25.6%) to the 2021–22 cohort, and 237 (19.6%) to the 2020–21 cohort; 826 (68.2%) are women and 385 (31.8%) are men; the average age is 18.3 years (SD = 1.8); 392 (32.4%) are from Experimental Sciences, 251 (20.7%) from Medicine, 157 (13.0%) from Health Sciences, 114 (9.4%) from the Advanced Polytechnic School, 111 (9.2%) from Communication Sciences, 138 (11.3%) from Law, Business and Government, and 48 (4.0%) from Education and Psychology.

The sample size was calculated using the pwr.t.test function from the pwr package (version 1.3–0) in R software (version 4.4.1), assuming a desired power of 0.80, an alpha level of 0.05, and an expected effect size of 0.30, which indicated a required sample of 89 paired observations. However, sampling was carried out by convenience, collecting paired responses from a total of 1,211 participants (the only faculty that presented a sample size below the desired threshold was Education and Psychology: *n* = 48).

### 2.3 Educational intervention

The UFV, aware of this challenge facing our society and our healthcare professionals, offers a humanities-based educational curriculum throughout the entire degree program. This is implemented through specific subjects for each academic year. Specifically, in the first year of undergraduate studies, there is a subject called *Skills and Competences of the Person*, also known as SCP, which aims to accompany each student both individually and as part of a community. This educational accompaniment is intended to favor “the full development of the student or learner, without being limited to academic achievement and without supplanting the learner himself as the subject of the action, with a non-directive and person-centered guidance model” (Crespí and López, 2023, p. 3).

This subject promotes the integral development and growth of students, impacting various areas of their lives: personal, academic, social, and professional by fostering transversal competences that are essential in any sphere of life. These competences enable individuals to become more fully themselves, more human, in any context, including professional practice. Its objectives include helping students: gain deeper self-knowledge, improve their social relationships (with classmates, colleagues, family members, etc.), and develop the transversal competences addressed in this subject, such as: proactivity, self-awareness, personal development, search for meaning in life: vocation and life project, teamwork, decision making, conflict resolution, project management: organization and planning, communication and leadership. All this, with the aim of helping them in their personal growth to reach the best version of themselves, to become who they are called to be and get on the way to their personal plenitude. To this end, the subject is structured around two learning environments:

1. Integral mentoring sessions, where students are individually guided by a mentor who specializes in educational accompaniment for their integral growth and development. The mentoring sessions specifically focus on the development of competences such as self-awareness, self-acceptance, personal growth, search for meaning in life, deep look, and proactivity. The program consists of six mentoring sessions in total: three during the first semester and three during the second semester, each lasting 1h.
2. The classroom, where students are accompanied as a community by their professor, an expert in developing transversal competences or soft skills, and by their peers. This is developed through a team project with social impact, using the PBL (Project-Based Learning) methodology. The teamwork component involves two key goals: (a) that the team is able to carry out a social improvement project with tangible results (*working as a team*), and (b) that both the team and its individual members experience growth and maturity from the moment the team is formed (*working within a team*). For this, the team and its members must put into practice the various competences targeted by the subject. Specifically, the classroom component focuses on developing the competences of cooperative work, work environment management, orientation towards results, verbal communication, paraverbal and non-verbal communication, and communication for encounter.

Table 1 summarizes the protocol of intervention of the subject (SCP).

**Table 1:** Protocol of intervention of the subject Skills and Competences of the Person (SCP).

Category	Personal skills and competencies (PSC)
Learning objective	Integral development and growth of students through transversal competences.
Learning Environments	6 individual mentoring sessions, each lasting 1 h. 34 classroom-based team project sessions, each lasting 1.5 h.
ECTS	5 ECTS.
Distribution of workload hours	Teacher-led activities: 50 h. Independent work: 75 h.
Competences	Mentoring: self-awareness, self-acceptance, self-management, search for meaning in life, orientation to excellence and proactivity. Classroom: teamwork: cooperative work, work environment management and orientation towards results. Effective communication: verbal communication, paraverbal and non-verbal communication, communication for encounter (empathy, assertiveness and listening).
Assessable learning activities	Individual assignments, team assignments, individual report on individual development, individual report on team development, individual oral presentation, team oral presentation, and theoretical competency-based exams.
Learning outcomes	Deepen self-knowledge. Identify strengths and weaknesses. Discern vocation. Create a personal development plan. Identify the consequences of their actions. Learn about different forms of conflict resolution. Improved interpersonal relationships. Give oral presentations using verbal and nonverbal language. Work on communication skills such as full presence, active listening, empathy and assertiveness. Work as a team using teamwork methodologies.
Facilitators	Mentors trained in educational accompaniment. Professors specialized in soft skills and transversal competences.
Methodology	Experiential learning and Project-Based Learning (PBL).

### 2.4 Measurement instrument

For this study, the BGCQ scale, The Basic Generic Competences Questionnaire (Crespí and García-Ramos, 2023), also known as the TPCQ, Transversal Personal Competences Questionnaire, was used. The BGCQ was specifically designed to assess transversal competences in university educational and professional contexts (Crespí and García-Ramos, 2023). Its structure and content are aligned with the conceptual framework of integral formation (Rodríguez Barroso et al., 2025), making it particularly suitable for evaluating the impact of programs aimed at developing intra- and interpersonal competences in higher education. The BGCQ has been previously applied in university contexts with same or similar pedagogical models (Crespí and López, 2023; Crespí et al., 2022; Crespí et al., 2025a, b).

This scale includes two key dimensions of transversal competences: (1) the intrapersonal dimension, which is divided into two subdimensions: (1.1) Deep Look, with 3 indicators (competences): self-awareness, self-acceptance, and self-management; and (1.2) Personal Development, with 3 indicators (competences): search for meaning in life, orientation to excellence, and proactivity; and (2) the interpersonal dimension, which is also divided into two subdimensions: (2.1) Teamwork, with 3 indicators (competences): cooperative work, work environment management, and orientation towards results; and (2.2) Effective Communication, with 3 indicators (competences): verbal communication, paraverbal and non-verbal communication, and communication for encounter. The structure of the questionnaire is presented in Table 2.

**Table 2:** BGCQ dimensional structure.

Dimension (D)	Subdimension (S)	Indicators (I) Competences (C)	Sub-indicators (SI)
Intrapersonal	Deep look	Self-awareness	Strengths
			Areas for improvement
			Distinctive personal characteristics
		Self-acceptance	Strengths and areas for improvement
			Unique and irreplaceable
			Being in constant development
		Self-management	Self-reliance
			Attribution of causality
			Responsibility
	Personal development	Search for meaning in life	Meaning of life
			Vocation
			Life project
		Orientation to excellence	Objectives of development
			Objectives involving a challenge
			Mentor or tutor
		Proactivity. Self-discipline	Action
			Overcoming obstacles
			Initiative
Interpersonal	Teamwork	Cooperative work	Involvement and engagement
			Attitude of service and support
			Integration into the team
		Work environment management	Politeness and respect
			Attitude
			Motivation
		Orientation towards results	Planning and organisation
			Assuming tasks
			Compliance with obligations
	Effective communication	Verbal communication	Key ideas
			Structure
			Clarity
		Paraverbal and non-verbal communication	Visual contact
			Body and hands
			Rhythm and tone
		Communication for encounter	Empathy
			Assertiveness
			Active listening

Fuente: Crespí y García-Ramos (2023, p. 5).

As indicated, each dimension has 2 subdimensions, each of which has 3 indicators (or competences), and each indicator includes 3 items, for a total of 36 items. A 6-point Likert scale is used (with 1 being the lowest rating and 6 the highest). Six response options are used to avoid central tendency bias. The BGCQ is included in Annex 1. This questionnaire has been validated in previous samples, thereby demonstrating its validity and reliability (Crespí and García-Ramos, 2023).

### 2.5 Data collection

Data collection was coordinated by one of the researchers involved in this study, who organized the collection of the entire sample during both the pre-test stage (at the beginning of each academic year: September 2020, September 2021, and September 2022) and the post-test stage (at the end of each academic year: May 2021, May 2022, and May 2023), across the three academic cohorts.

The classroom instructors of the SCP subject collaborated by reading the same instructions to all students and providing a QR code to access the questionnaire (via Google Forms). From the outset, each participant was identified with an alphanumeric code, which made it possible to link each individual’s responses across the different time points.

### 2.6 Data analysis

The categorical variables collected, such as faculty, gender or cohort, were described using frequencies and percentages, while quantitative variables, such as age or questionnaire scores, were expressed by mean and standard deviation. The percentage of missing values was analyzed, and imputation was performed using the Random Forest technique (Machine Learning).

Globally and using a multivariate approach, an attempt was made to estimate a generalized linear model to study the effect of time (before and after the subject) and other covariates on the score of each dimension, using the Gamma distribution, random slopes and intercepts for the faculty variable, and a possible interaction between the time variable and the faculty variable. However, none of these models were successfully identified in any of their forms and/or presented convergence issues.

Therefore, two linear mixed models were estimated to predict scores in the intrapersonal and interpersonal dimensions, assuming the effect of the following covariates as fixed effects: age, gender and faculty of each student. The ‘lmerTest’ function (Kuznetsova et al., 2017), version 3.1–3, of the R software (version 4.4.1), was used for this analysis.

Specifically, the differences in scores for each dimension, subdimension, and competence between the pre-intervention and post-intervention time points were analyzed using the paired-samples Student’s t-test and Cohen’s *d* effect size index (considered small if less than 0.5, moderate between 0.5 and 0.8, and large if greater than 0.80, according to Cohen, 1988). It was used the cohens_d function from the rstatix package (version 0.7.2.) in R. This analysis was conducted for the entire sample (both imputed and non-imputed, as a sensitivity analysis) and separately for each faculty and cohort. The *post hoc* paired t test power calculation was performed by pwr package (version 1.3–0) in R.

Comparisons between faculties and cohorts for each score were conducted using one-way ANOVA, the η^2^ effect size index (according to Cohen, 1988: values of approximately 0.01, 0.06, and 0.14 indicate small, medium, and large effects, respectively), and Dunn’s post-hoc comparisons along with the Benjamini and Hochberg (BH) correction method to examine differences between each pair of groups. The η^2^ effect size index was estimated by the etaSquared function from the lsr package (version 0.5.2.) in R.

Differences in scores between men and women were also analyzed. These score comparisons between the described groups were performed for both the scores obtained at each time point and the differences between the two time points. The Type I error assumed for all analyses was 0.05.

### 2.7 Ethical considerations

Students voluntarily participated in the completion of the BGCQ questionnaire. Explicit informed consent was obtained, clearly stating that the data collected would be used exclusively for research purposes. Anonymity of responses and confidentiality of data were ensured in accordance with the General Data Protection Regulation (European Union, 2016), the Spanish Organic Law 3/2018 on the Protection of Personal Data and Guarantee of Digital Rights, and the university’s internal data protection policies.

Participants were also informed of their right to withdraw consent at any time without any negative consequences. The study was conducted in full compliance with the ethical principles outlined in the Declaration of Helsinki, as well as the established guidelines for research in the social sciences and humanities. No sensitive personal data were collected, and no procedures or interventions were implemented that could entail any physical, psychological, or social risk to participants.

## 3 Results

### 3.1 Descriptive analyses

Table 3 presents the descriptive analyses showing the characteristics of the students participating in the study, according to faculty. These analyses indicate that: (1) the largest sample comes from the Faculty of Experimental Sciences (392 students, 32.37%), followed by Medicine (251 students, 20.73%), Health Sciences (157 students, 12.96%), and then the remaining faculties; (2) the majority of students are women (826 students, 68.21%), both overall and within each faculty, except for the Polytechnic School, where there are 30 women (26.3%); the average age is 18.8 years (SD = 1.8).

**Table 3:** Descriptive variables of participants by faculty.

Sociodemographic variables	Overall, *N* = 1,211^1^	Experimental Sciences, *N* = 392^1^	Communication Sciences, *N* = 111^1^	Health Sciences, *N* = 157^1^	Law, Business and Government, *N* = 138^1^	Education and Psychology, *N* = 48^1^	Medicine, *N* = 251^1^	Advanced Polytechnic School, *N* = 114^1^
Cohort								
22–23	664 (54.8%)	173 (44.1%)	77 (69.4%)	93 (59.2%)	89 (64.5%)	44 (91.7%)	106 (42.2%)	82 (71.9%)
21–22	310 (25.6%)	146 (37.2%)	22 (19.8%)	16 (10.2%)	28 (20.3%)	0 (0.0%)	80 (31.9%)	18 (15.8%)
20–21	237 (19.6%)	73 (18.6%)	12 (10.8%)	48 (30.6%)	21 (15.2%)	4 (8.3%)	65 (25.9%)	14 (12.3%)
Gender								
Woman	826 (68.2%)	289 (73.7%)	78 (70.3%)	80 (51.0%)	93 (67.4%)	39 (81.3%)	217 (86.5%)	30 (26.3%)
Man	385 (31.8%)	103 (26.3%)	33 (29.7%)	77 (49.0%)	45 (32.6%)	9 (18.8%)	34 (13.5%)	84 (73.7%)
AGE (post)								
Mean (SD)	18.8 (1.8)	18.5 (0.8)	19.0 (1.3)	20.0 (3.4)	18.8 (1.7)	19.9 (2.8)	18.5 (0.8)	18.8 (1.5)
Median [25–75%]	18.0 [18.0–19.0]	18.0 [18.0–19.0]	19.0 [18.0–19.0]	19.0 [18.0–20.0]	18.0 [18.0–19.0]	19.0 [18.0–20.0]	18.0 [18.0–19.0]	18.0 [18.0–19.0]
Range	18.0, 37.0	18.0, 24.0	18.0, 24.0	18.0, 37.0	18.0, 35.0	18.0, 31.0	18.0, 24.0	18.0, 28.0

The percentage of missing responses across the entire database was 3.622%. A total of 41.16% of participants had at least one missing value in their responses to one or more items. The five items with the highest rates of missing responses were: item 25 (with 14.2% missing), item 32 (10.9%), item 33 (10.9%), item 3 (10.7%), and item 31 (10.4%). The faculties most affected by this missing data were Experimental Sciences, followed by Communication, Law, Business and Government, and the Polytechnic School.

The imputation process was computationally satisfactory, with estimated imputation errors below 1 for all items with the exception of items 10 (SE = 1.335), 15 (SE = 1.418), and 25 (SE = 1.135). Statistically, the change in scores after the educational intervention was significant in both dimensions of the questionnaire (*p* < 0.001), both before and after the imputation of missing values. However, the effect size was slightly lower in the non-imputed case (intrapersonal dimension: Cohen’s *d* = 0.086; interpersonal dimension: Cohen’s *d* = 0.095) compared to the imputed data (Cohen’s *d* = 0.146; *d* = 0.346, respectively). To ensure the highest possible statistical power and to minimize potential convergence issues in the estimated models, the imputed sample was used for the subsequent analyses.

### 3.2 Differences in scores at different moments

As shown in Table 4, the pre-test and post-test mean scores for each dimension, subdimension, and competence. For the entire student body, the intrapersonal dimension shows a greater average improvement between the pre and post moments (Cohen’s *d* = 0.349) than the interpersonal dimension (Cohen’s *d* = 0.146), although in both cases the results are significant (*p* < 0.001). Significant differences are thus observed for the intra- and interpersonal dimensions, the subdimensions of deep look, personal development and effective communication, as well as their respective competences: self-awareness, self-acceptance, self-management, search for meaning in life, orientation to excellence, proactivity, verbal communication, paraverbal and non-verbal communication, and communication for encounter. No significant differences were found for the teamwork subdimension, nor for their respective competences: cooperative work, work environment management and orientation towards results. Regarding effect size, differences in the average scores of slight magnitude are observed, with effect size indices ranging from 0.114 (communication for encounter) to 0.499 (self-awareness).

**Table 4:** Self-perceived development of transversal, intra and interpersonal competences.

Dimensions (D) Subdimensions (S) Competences (C)	Overall, *N* = 2,422^1^	PRE-TEST, *N* = 1,211^1^	95% CI^2^	POST-TEST, *N* = 1,211^1^	95% CI^2^	*p* ^3^	Cohen’s *d*
Intrapersonal (D)	88.5 (9.1)	86.9 (8.5)	86, 87	90.0 (9.4)	89, 91	**<0.001**	−0.349
Interpersonal (D)	91.6 (8.9)	91.0 (8.3)	91, 91	92.3 (9.4)	92, 93	**<0.001**	−0.146
Deep look (S)	43.9 (5.1)	42.9 (4.8)	43, 43	44.8 (5.2)	45, 45	**<0.001**	−0.387
Personal development (S)	44.6 (5.1)	44.0 (4.9)	44, 44	45.2 (5.2)	45, 45	**<0.001**	−0.239
Teamwork (S)	46.9 (4.8)	46.9 (4.4)	47, 47	46.9 (5.1)	47, 47	0.7	0.010
Effective communication (S)	44.7 (5.1)	44.0 (4.8)	44, 44	45.4 (5.2)	45, 46	**<0.001**	−0.268
Self-awareness (C)	13.9 (2.1)	13.4 (2.0)	13, 14	14.4 (2.1)	14, 15	**<0.001**	−0.499
Self-acceptance (C)	14.9 (2.3)	14.6 (2.3)	14, 15	15.2 (2.3)	15, 15	**<0.001**	−0.261
Self-management (C)	15.1 (1.9)	14.9 (1.8)	15, 15	15.2 (2.0)	15, 15	**<0.001**	−0.174
Search for meaning in life (C)	15.1 (2.3)	14.9 (2.4)	15, 15	15.4 (2.3)	15, 15	**<0.001**	−0.205
Orientation to excellence (C)	14.6 (2.1)	14.4 (2.0)	14, 14	14.8 (2.2)	15, 15	**<0.001**	−0.181
Proactivity (C)	14.9 (2.0)	14.7 (2.0)	15, 15	15.1 (2.0)	15, 15	**<0.001**	−0.170
Cooperative work (C)	15.8 (2.0)	15.8 (1.8)	16, 16	15.7 (2.1)	16, 16	0.2	0.037
Work environment management (C)	15.9 (1.7)	15.9 (1.6)	16, 16	16.0 (1.8)	16, 16	0.2	−0.045
Orientation towards results (C)	15.2 (2.0)	15.2 (1.8)	15, 15	15.2 (2.1)	15, 15	0.4	0.027
Verbal communication (C)	15.4 (1.8)	15.1 (1.7)	15, 15	15.6 (1.9)	16, 16	**<0.001**	−0.280
Paraverbal and non-verbal (C)	14.0 (2.7)	13.6 (2.6)	13, 14	14.3 (2.8)	14, 14	**<0.001**	−0.232
Communication for encounter (C)	15.4 (1.9)	15.3 (1.8)	15, 15	15.5 (1.9)	15, 16	**<0.001**	−0.114

^1^n (%); Mean (SD); Median [IQR]; ^2^CI = Confidence Interval; ^3^Paired t-test. Bold *p*-values indicate statistically significant differences (*p* < 0.05).

**Figure 1:**
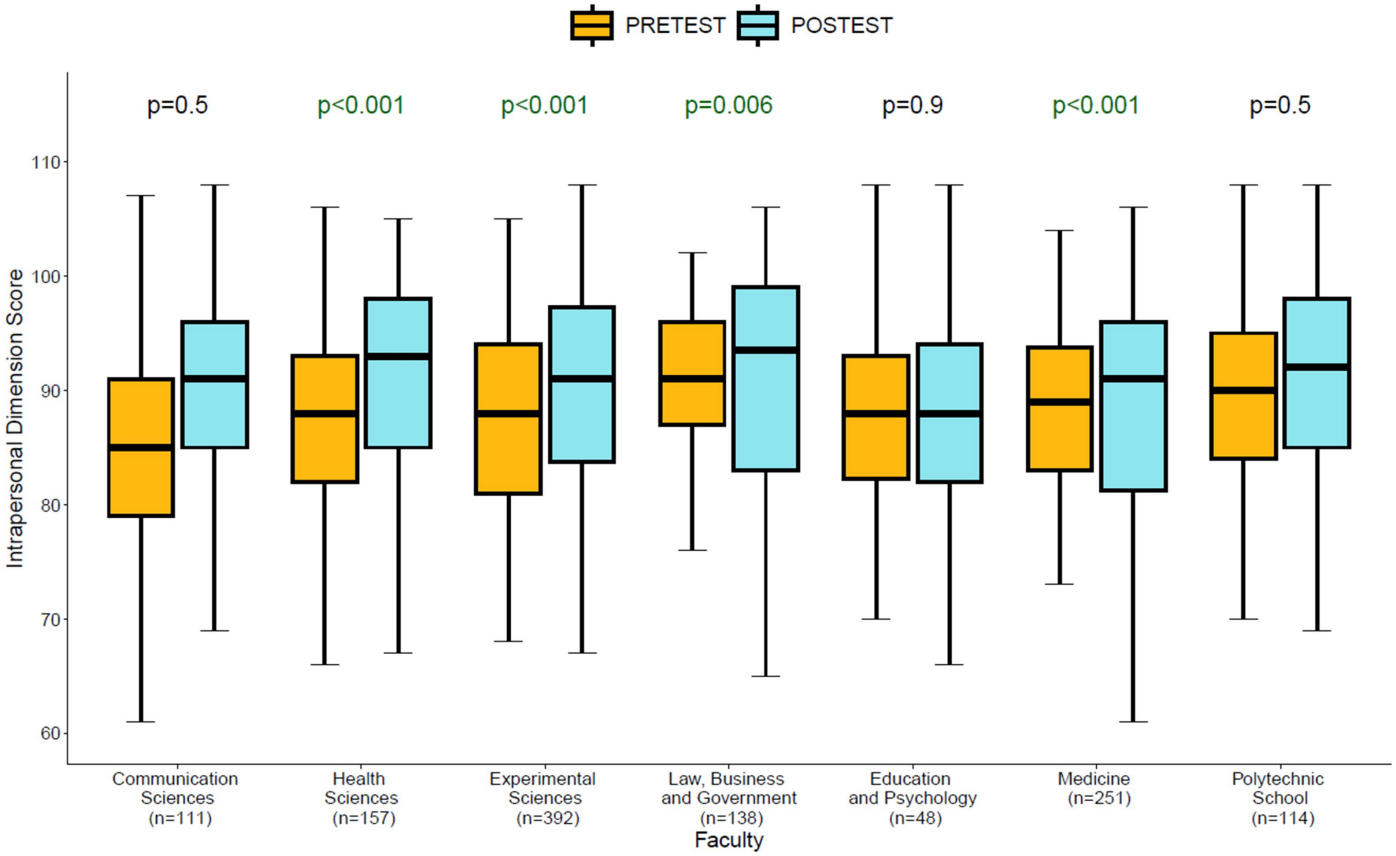
Significant differences pre-test/post-test for intrapersonal dimensions by faculty.

When analyzing the differences between the pre-test and post-test moments for intrapersonal dimension across the different faculties (Figure 1), significant differences in the self-perception of intrapersonal development are observed in favor of the post-intervention moment (post-test) in the Faculties of Health Sciences (*p* < 0.001), Experimental Sciences (*p* < 0.001), Law, Business and Government (*p* = 0.006), and Medicine (*p* < 0.001).

The average of each score for each faculty is shown in Annex 2.

**Figure 2:**
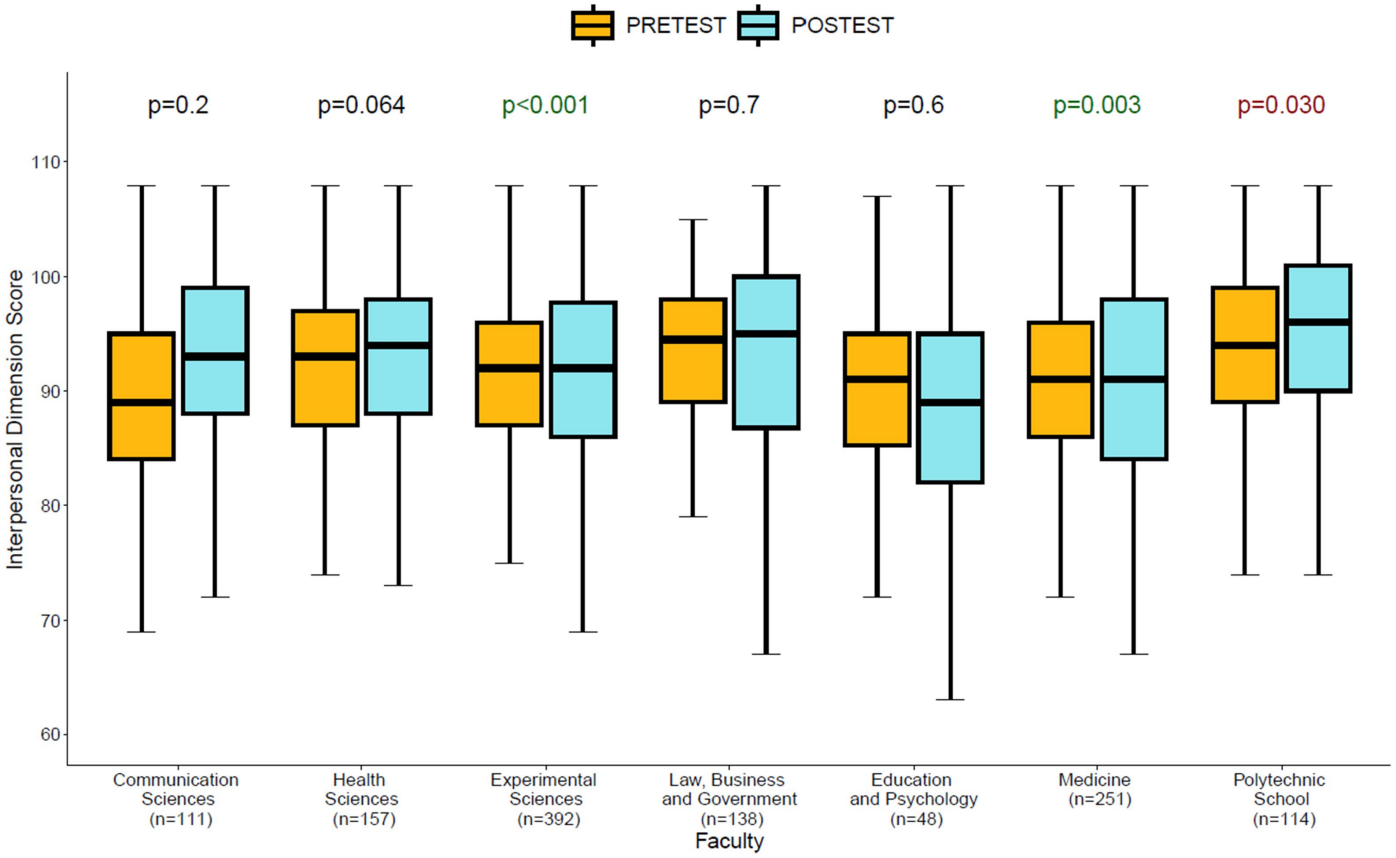
Significant differences pre-test/post-test for interpersonal dimensions by faculty.

The differences between the pre-test and post-test moments for the interpersonal dimension across the different faculties (Figure 2) show significant differences in the self-perception of interpersonal development in favor of the post-intervention moment (post-test) in the Faculties of Experimental Sciences (*p* < 0.001) and Medicine (*p* = 0.003). The only faculty that shows significantly lower self-perception scores after the intervention (Mean = 87.7, SD = 11.5) compared to the pre-test moment (Mean = 90.4, SD = 7.8) for the interpersonal dimension is the Advanced Polytechnic School (*p* = 0.030). The remaining faculties do not show a significant change in the average scores (see Figure 2).

The *post hoc* estimated power of the paired-sample comparison across the whole university was 1 − *β* = 0.99 (using the observed effect size obtained for the differences in the interpersonal dimension: *d* = 0.146 and the global sample size: *n* = 1,211). The power for the differences in this same dimension ranged from the faculty with the smallest sample size (Education and Psychology; *n* = 48; *d* = 0.08; power 1 − β = 0.10) to the faculty with the largest sample size (Experimental Sciences; *n* = 395; *d* = 4.05; power 1 − β = 0.99).

### 3.3 Multivariant linear mixed models for each dimension

Regarding the two estimated linear mixed models for each dimension, a significant effect (*p* < 0.001) of the passage of time (pre–post educational intervention) on the scores was observed (Table 5). Controlling for the partial effects of covariates such as age, gender, and faculty, it is predicted that at the post-intervention moment, the score in the interpersonal dimension will be 1.319 points higher than at the pre-intervention moment, and 2.914 points higher in the case of the intrapersonal dimension.

**Table 5:** Self-perceived development of intra and interpersonal competences.

	Interpersonal dimension				Intrapersonal dimension			
	Beta	SE^1^	95% CI^1^	*p*	Beta	SE^1^	95% CI^1^	*p*
(Intercept)	88.9836	2.40	84.2782, 93.6890	**<0.001**	77.0016	2.45	72.1973, 81.8058	**<0.001**
Time period								
PRE-TEST	—	—	—	—	—	—	—	—
POST-TEST	1.3189	0.261	0.8067, 1.8312	**<0.001**	2.9139	0.268	2.3873, 3.4405	**<0.001**
Faculty								
Communication Sciences	—	—	—	—	—	—	—	—
Health Sciences	2.1023	0.937	0.2644, 3.9403	**0.025**	0.9835	0.956	−0.8923, 2.8593	0.304
Experimental Sciences	0.1919	0.803	−1.3829, 1.7667	0.811	−0.6874	0.819	−2.2946, 0.9198	0.402
Law, Business and Government	0.7624	0.949	−1.0995, 2.6242	0.422	0.8782	0.969	−1.0220, 2.7784	0.365
Education and Psychology	2.4191	1.29	−0.1124, 4.9505	0.061	2.3235	1.32	−0.2600, 4.9070	0.078
Medicine	3.3641	0.854	1.6880, 5.0403	**<0.001**	2.2589	0.872	0.5482, 3.9695	**0.010**
Advanced Polytechnic School	−0.2370	1.02	−2.2295, 1.7556	0.816	0.5839	1.04	−1.4497, 2.6174	0.573
Gender								
Man	—	—	—	—	—	—	—	—
Woman	2.6254	0.494	1.6563, 3.5945	**<0.001**	1.4119	0.504	0.4229, 2.4010	**0.005**
Age	−0.0542	0.123	−0.2962, 0.1878	0.661	0.4525	0.126	0.2054, 0.6996	**<0.001**

Multivariable linear mixed models. ^1^SE = Standard Error, CI = Confidence Interval. Bold *p*-values indicate statistically significant differences (*p* < 0.05).

On the other hand, for the interpersonal dimension, it is predicted that the scores of the female group are 2.625 points higher than those of the male group (*p* < 0.001), as well as scores from the Faculty of Medicine being 3.364 points higher (*p* < 0.001), and those from the Faculty of Health Sciences 2.102 points higher (*p* = 0.025), compared to the Faculty of Communication Sciences (reference category). For the intrapersonal dimension, a significant effect is predicted on the scores associated with being female (beta = 1.412; *p* = 0.005), with age (beta = 0.452; *p* < 0.001), and with belonging to the Faculty of Medicine (beta = 2.259; *p* = 0.010).

### 3.4 Differences between groups: faculties and cohorts

The analyses carried out show significant differences in scores between faculties at the pre-test, post-test, and in progress (difference between moments). The faculties that scored highest at the pre-test moment in the intrapersonal dimension were: Education and Psychology (mean score, *M* = 90.9; standard deviation, SD = 7.3), Medicine (*M* = 89.0, SD = 7.9), Advanced Polytechnic School (*M* = 87.6, SD = 8.5), and Communication Sciences (*M* = 87.6, SD = 8.5), followed by Health Sciences (*M* = 87.4, SD = 8.0), Experimental Sciences (*M* = 84.2, SD = 8.4), and lastly, Business, Law and Government (*M* = 47.3, SD = 4.1). Regarding the interpersonal dimension, the faculties with the highest scores were: Medicine (*M* = 93.6, SD = 7.3), Education and Psychology (*M* = 92.8, SD = 8.7), Health Sciences (*M* = 91.4, SD = 8.6), Communication Sciences (*M* = 91.0, SD = 8.2), followed by the Advanced Polytechnic School (*M* = 90.4, SD = 7.8), Experimental Sciences (*M* = 89.0, SD = 8.6), and finally, Business, Law and Government (*M* = 43.7, SD = 4.9). The faculties that scored highest at the post-test moment in the intrapersonal dimension were: Medicine (*M* = 91.3, SD = 8.7), Health Sciences (*M* = 90.8, SD = 8.7), Education and Psychology (*M* = 90.7, SD = 10.3), Experimental Sciences (*M* = 90.0, SD = 9.0), followed by Business, Law and Government (*M* = 89.8, SD = 10.6), Communication Sciences (*M* = 88.2, SD = 10.8), and finally, the Advanced Polytechnic School (*M* = 88.1, SD = 9.6). Regarding the interpersonal dimension, the faculties with the highest scores were: Medicine (*M* = 94.9, SD = 8.0), Education and Psychology (*M* = 93.5, SD = 9.3), Health Sciences (*M* = 92.7, SD = 8.4), Experimental Sciences (*M* = 92.6, SD = 8.9), followed by Business, Law and Government (*M* = 91.3, SD = 9.1), Communication Sciences (*M* = 89.9, SD = 10.7), and finally, the Advanced Polytechnic School (*M* = 87.7, SD = 11.5).

The faculties whose difference in scores (mean difference: ΔM) between moments represented positive progress in the intrapersonal dimension were: Experimental Sciences (ΔM = 5.8, SD = 9.0), Health Sciences (ΔM = 3.4, SD = 8.8), followed by Medicine (ΔM = 2.3, SD = 8.3), Business, Law and Government (ΔM = 2.2, SD = 9.1), Communication Sciences (ΔM = 0.6, SD = 9.4), and Advanced Polytechnic School (ΔM = 0.5, SD = 8.4). Only the Faculty of Education and Psychology (ΔM = −0.2, SD = 9.5) showed negative progress between the two moments. Regarding the interpersonal dimension, the faculties that showed positive progress were: Experimental Sciences (ΔM = 3.5, SD = 8.9), Health Sciences (ΔM = 1.3, SD = 8.7), Medicine (ΔM = 1.3, SD = 6.9), followed by Education and Psychology (ΔM = 0.7, SD = 10.3), and Business, Law and Government (ΔM = 0.3, SD = 8.6). The following faculties showed a decline in their averages after the intervention: Communication Sciences (ΔM = −1.1, SD = 9.4) and the Advanced Polytechnic School (ΔM = −2.7, SD = 9.7).

**Figure 3:**
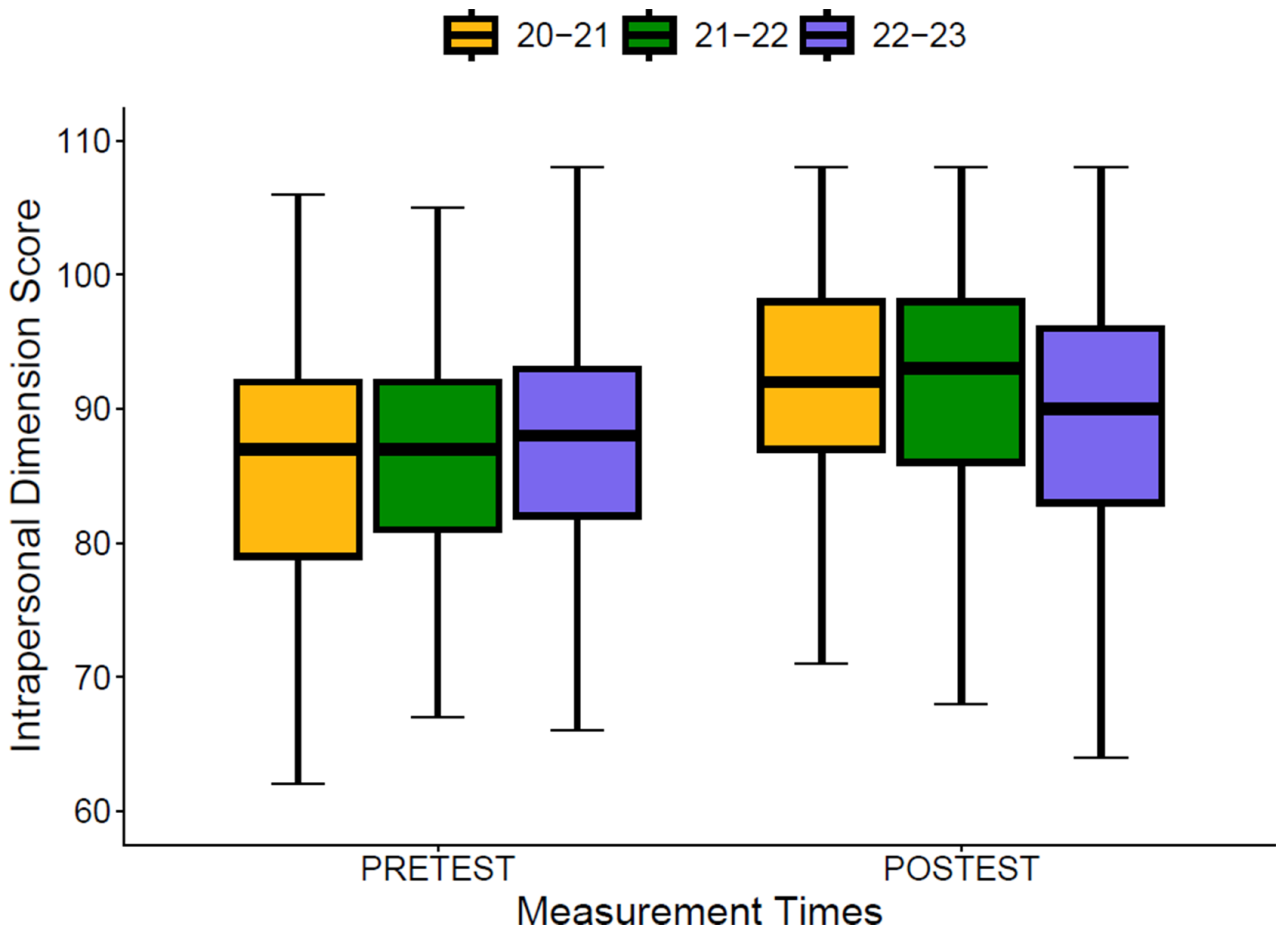
Differences between cohorts for the intrapersonal dimension.

**Figure 4:**
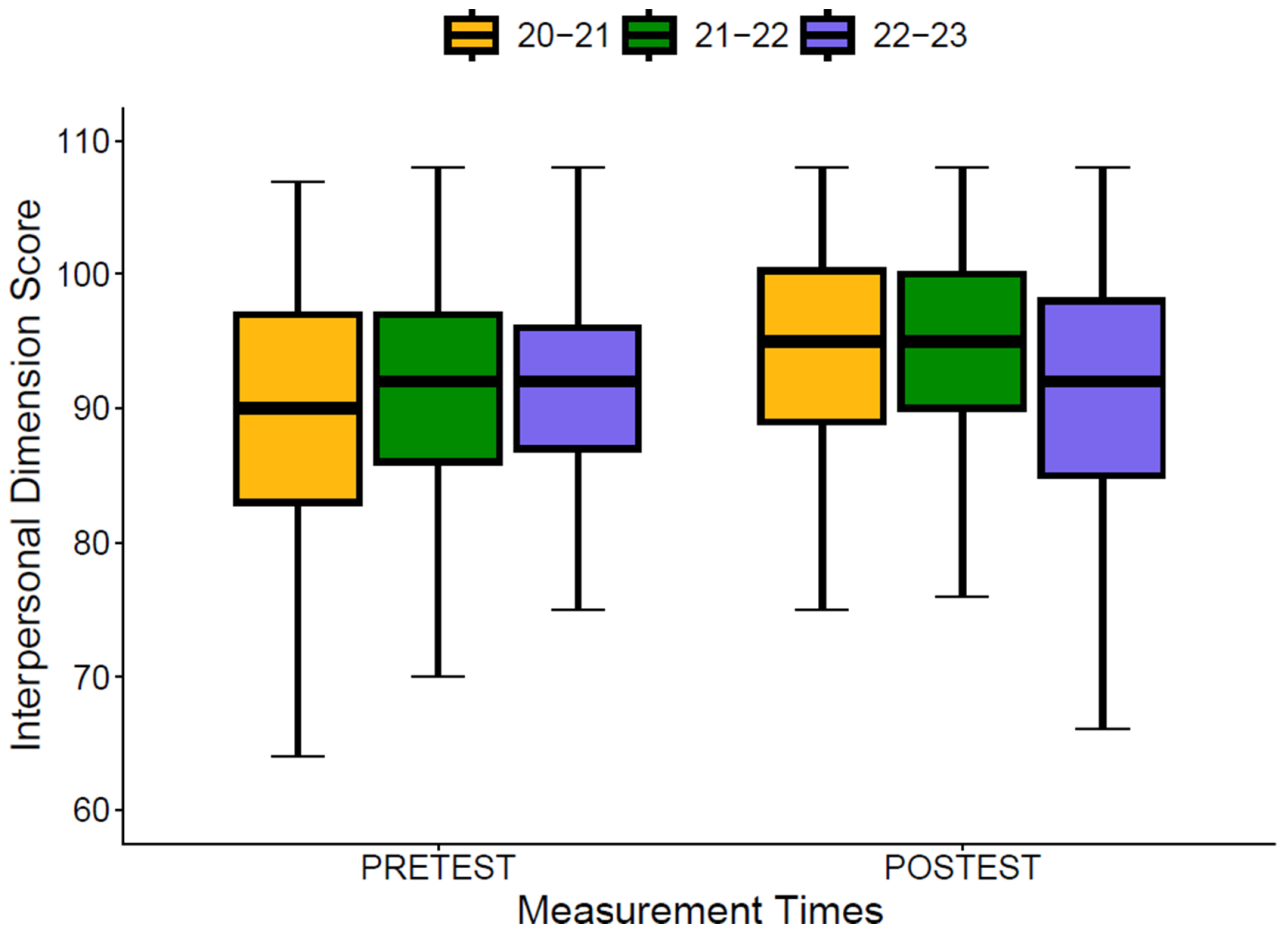
Differences between cohorts in the interpersonal dimension.

Regarding the differences between cohorts at the pre-test and post-test moments (Figures 3, 4), significant differences were found at the pre-test moment for the Intrapersonal Dimension (*p* = 0.040; Eta Squared = 0.005) and the Interpersonal Dimension (*p* < 0.001; Eta Squared = 0.016), with a small effect size. The same occurs at the post-test moment for the Intrapersonal Dimension (*p* < 0.001; Eta Squared = 0.023) and the Interpersonal Dimension (*p* < 0.001; Eta Squared = 0.045). Regarding progress, significant differences between cohorts were also found for the Intrapersonal Dimension (*p* < 0.001; Eta Squared = 0.049) and for the Interpersonal Dimension (*p* < 0.001; Eta Squared = 0.087), although in this case the effect size is moderate.

## 4 Discussion

In healthcare, the term *humanization* typically refers to an integral and holistic approach to patient care (Henao-Castaño et al., 2021), involving an integrated consideration of the biological, psychological, social, and behavioral dimensions of the individual (Maestre, 2013; Ramos-Brieva et al., 2017). The lack of humanization in healthcare is the result of a complex series of interrelated and mutually influencing factors. These elements stem, in part, from the broader social framework and the ways in which healthcare institutions are structured and operate (March, 2017). Contributing factors include the lack of personalized and high-quality care, often caused by work overload and the mechanization of patient care, the absence of empathy resulting from stress and prolonged exposure to patient pain and suffering (Condori Salluco and Choque Rojas, 2022; Jiménez-Rodríguez et al., 2019), moral disengagement (Jiménez-Rodríguez et al., 2019), and insufficient training in competences such as compassion (Henao-Castaño et al., 2021) and communication (Condori Salluco and Choque Rojas, 2022; Reyes-Téllez et al., 2024). These factors can hinder healthcare professionals’ ability to establish an effective and human connection with their patients. Additionally, an overreliance on technology contributes to the deterioration of the doctor–patient relationship, leading to emotional distancing and a progressive loss of trust (Condori Salluco and Choque Rojas, 2022). Moreover, these shortcomings are reflected in daily clinical practice and affect respect for patient privacy, autonomy, and rights (March, 2017; Ley 24/ 2002 del 14 de noviembre).

To address these challenges, it is essential to consider the formative dimension of healthcare professionals. The lack of humanization in clinical practice is closely linked to gaps in training, particularly in the development of transversal competences. Providing high-quality, integral training that enables professionals to deliver excellent care is considered a best practice in promoting humanization (Heras La Calle, 2017). This connection between professional behavior and educational background highlights the need for university programs that intentionally foster humanistic attitudes and interpersonal skills from the outset of professional training. Specifically, the humanization of healthcare is closely linked to the training and development of soft skills (Henao-Castaño et al., 2021), with communication playing a particularly significant and impactful role (Condori Salluco and Choque Rojas, 2022; Gonçalves de Oliveira et al., 2006; Jiménez-Rodríguez et al., 2019). Soft skills, also referred to as generic or transversal competences, are highly in demand and essential in professional practice (Garavito-Hernández et al., 2024; Tang, 2019; Tripathy, 2021). These competences are also emphasized in the context of healthcare professionals (Vargas and Zaldivar, 2023), including those in medicine (D’Ottavio, 2023) and nursing (Álvarez et al., 2024). In Europe, leading organizations such as the AMEE (Association for Medical Education in Europe) and, in Spain, the Sociedad Española de Educación Médica (SEDEM), stress the importance and necessity of developing such competences [Sandars et al., 2014; Sociedad Española de Educación Médica (SEDEM), and Sociedad Española de Medicina Interna (SEMI), 2025]. In this regard, professional socialization plays a key role in shaping healthcare professionals’ identity and transversal competences, through both formal education and the influence of role models such as mentors and clinical teachers (Jawula Salisu et al., 2019; Moon and Chang, 2023).

With regard to the training and development of these types of competences in higher education, they are typically integrated into technical or discipline-specific subjects, most often through group work (Mwita et al., 2023; Tang, 2019), and more specifically, through active learning methodologies such as project-based learning (Fernández de Caleya et al., 2023), problem-based learning (Deep et al., 2019), and project-oriented learning (Crespí et al., 2022). Other approaches to fostering these competences include specialized training workshops (Yan et al., 2019), elective or complementary subjects (Villardón-Gallego, 2015), extracurricular activities (Feraco et al., 2023), the adoption of innovative educational strategies within the learning process (Pluzhnirova et al., 2021), mentoring or coaching (Crespí and López, 2023; Tang, 2019), game-based learning (García et al., 2020), and role-playing activities (Tang, 2019). What is far less common, however, is the inclusion of mandatory curricular subjects specifically designed to train these competences within study programs, as well as the availability of scientific evidence regarding their development. For this reason, the present study describes an original and innovative initiative that introduces a mandatory curricular subject aimed at the development of soft skills in higher education. Specifically, it focuses on the subject Integral Formation: Skills and Competences of the Person (SCP), as an educational intervention aimed at developing transversal competences that contribute to the humanization of professionals in the healthcare field. The SCP subject offers a structured curricular program that provides integral accompaniment, both individually, through mentoring, and collectively, within the classroom, aimed at first-year students from various degree programs across different faculties, including those in healthcare. The main objective was to assess the development of intra- and interpersonal competences before and after the educational intervention, using a validated self-report scale (Crespí and García-Ramos, 2023).

In this way, this research aims to study the formative impact of SCP on the self-perception of development of the intrapersonal transversal competences: self-awareness, self-acceptance, self-management (deep look subdimension), search for meaning in life, orientation to excellence, proactivity (personal development subdimension) and interpersonal transversal competences: cooperative work, work environment management, orientation towards results (teamwork subdimension), verbal communication, paraverbal and non-verbal communication and communication for encounter (effective communication subdimension), in university students, with a special focus on health sciences students.

The research findings indicate that, across the entire student sample from all faculties, the intrapersonal dimension showed a greater average improvement between the pre- and post-intervention assessments (Cohen’s *d* = 0.349) than the interpersonal dimension (Cohen’s *d* = 0.146), although both changes were statistically significant (*p* < 0.001). A multivariate analysis also revealed a significant and positive effect on scores in both dimensions (*p* < 0.001). These results are consistent with previous similar studies (Crespí, 2018; Crespí et al., 2025a, b). This study contributes further by disaggregating results by faculty, with particular attention given to Health Sciences, Medicine, and Experimental Sciences. Overall, the results suggest that the educational intervention (the SCP subject) is effective in improving students’ self-perception of soft skills development, skills that are key to the formation of professionals as agents of humanization within the healthcare system. Specifically, students appear to perceive the greatest development in the competences most in demand and most essential for professional practice in the healthcare field. These include: self-awareness (Cohen’s *d* = 0.499), self-acceptance (Cohen’s *d* = 0.261), within the deep look subdimension, search for meaning in life (Cohen’s *d* = 0.205), within the personal development subdimension, verbal communication (Cohen’s *d* = 0.280), Paraverbal and non-verbal communication (Cohen’s *d* = 0.232), both within the effective communication subdimension.

In this regard, Jiménez-Rodríguez et al. (2019) identify as a critical aspect of dehumanization the failure to see the person as a whole, instead “focusing on a part of the body without considering the entire organism and the patient’s mental states” (p. 87). In this sense, the subject appears to have had a notable impact on students’ perceived development of the deep look competence (Cohen’s *d* = 0.387), which refers to the ability to view others, oneself, and reality with all their possibilities, not only as they are now, but also in terms of what they may become. This is a perspective that acknowledges human dignity, viewing each person as valuable, unique, and unrepeatable, with infinite potential. Jiménez-Rodríguez et al. (2019) also highlight manifestations of dehumanization in non-verbal behaviors such as interactional distance, eye contact, and paraverbal aspects like speech rate and tone, which can communicate rejection. In this regard, the subject also appears to have had an impact on the development of *communication* skills, not only in terms of verbal language, but also in paraverbal and non-verbal aspects (Cohen’s *d* = 0.232). Among the competences most often cited as lacking among healthcare professionals are communication (Condori Salluco and Choque Rojas, 2022; García-Salido et al., 2019; March, 2017), and more specifically, empathy (Condori Salluco and Choque Rojas, 2022; Jiménez-Rodríguez et al., 2019) and compassion (Henao-Castaño et al., 2021). Regarding the communication for encounter competence, within which the SCP subject develops empathy, assertiveness, and active listening, students also reported an increase in perceived development after completing the subject (Cohen’s *d* = 0.114). These are communication skills considered “key for students to acquire during their academic training” (D’Ottavio, 2023, p. 87).

On the other hand, when examining the progression within each faculty, that is, the differences between the pre- and post-intervention assessments, it is evident that the faculties showing the greatest improvement, surpassing the other faculties at UFV, are the Faculty of Experimental Sciences, with statistically significant differences in both dimensions (*p* < 0.001); the Faculty of Medicine, with significant differences in both the intrapersonal (*p* < 0.001) and interpersonal (*p* = 0.003) dimensions; and the Faculty of Health Sciences, with a significant difference in the intrapersonal dimension (*p* < 0.001). When comparing the degree of progress (difference between pre- and post-intervention) across faculties, the data show that, for both the intrapersonal and interpersonal dimensions, the Faculty of Experimental Sciences reported the most substantial gains (Intrapersonal: ΔM = 5.8, SD = 9.0; Interpersonal: ΔM = 3.5, SD = 8.9), followed by the Faculty of Health Sciences (Intrapersonal: ΔM = 3.4, SD = 8.8; Interpersonal: ΔM = 1.3, SD = 8.7), and the Faculty of Medicine (Intrapersonal: ΔM = 2.3, SD = 8.3; Interpersonal: ΔM = 1.3, SD = 6.9). These were followed by the remaining faculties.

Regarding potential differences between cohorts, both at the pre-test and post-test stages, statistically significant differences were observed, with effect sizes ranging from small to moderate, and therefore considered to be of limited practical relevance.

In contrast, being over 18 years old, identifying as female, and belonging to the Faculties of Medicine or Health Sciences were all associated with significantly higher scores in one or both dimensions. Specifically, students from the Faculty of Medicine scored significantly higher than students from other faculties in both the intrapersonal (*p* = 0.010) and interpersonal (*p* < 0.001) dimensions. Similarly, students from the Faculty of Health Sciences also scored significantly higher than their peers in the interpersonal dimension (*p* = 0.025).

## 5 Conclusion

The main conclusions of this study are as follows: (1) university curricular subjects focused on skills and competences appear to be effective in fostering their development; (2) the competences with the greatest perceived development are associated with the subdimensions of deep look, personal development, and effective communication; (3) the competences with the least perceived development are linked to the teamwork subdimension, likely due to a high initial self-perception, which leaves less room for perceived improvement. This is further compounded by the complexity involved in developing this competence, an issue highlighted by students who noted that working in a group is not the same as working as a team (Szalados, 2021).

This study provides findings related to a unique and innovative initiative within the educational landscape of health sciences education, aimed at promoting the development of soft skills to foster the humanization of healthcare systems. Several official bodies (such as the OECD, EHEA, AMEE, and SEDEM), as well as scientific studies cited in this article (e.g., Palés-Argullós and Nolla-Domenjó, 2016; Vargas and Zaldivar, 2023; Martínez Clares and González Morga, 2018; Munuera Gómez and Navarro Asencio, 2015; Reyes-Téllez et al., 2024; Saravia Domínguez et al., 2024), support the promotion of continuous training in soft skills at the university level to advance “the humanization of healthcare as a guiding axis for policy improvement and quality of care, as well as in the cohort of practices that facilitate collaborative work, communication, and human development” (Henao-Castaño et al., 2021, p. 84). The results of this study, together with the curricular educational proposal of the SCP subject, may serve as a valid and transferable example for other university curricular contexts.

## 6 Limitations and prospectives

This study presents a number of limitations:

The use of a self-report scale restricts the assessment of competence acquisition to a single perspective. However, the use of pre- and post-intervention measurements helps to minimize potential bias. While the use of the BGCQ, an instrument for assessing transversal competences with demonstrated validity and reliability, is valuable, it would have been appropriate to complement it with other measurement methodologies, such as 360° assessments (incorporating feedback from peers and faculty), behavioral evidence through role-plays or practical cases, in-depth interviews, etc. Furthermore, this scale has not been validated against a criterion measure that evaluates the predictive capacity of the scores in relation to actual competence-related behaviors. Additionally, since the evaluation was conducted at the beginning of the students’ academic careers, it is not possible to determine whether the self-perception of progress increases or decreases over time, nor what factors influence such changes. In this regard, further research is needed to assess the long-term effectiveness of this type of intervention. Moreover, as Van der Vleuten (2015) points out, the acquisition of these competences, which involve complex behaviors, cannot be fully achieved within a limited timeframe followed by an assessment. They require both sustained development and integration throughout the curriculum.
Another limitation lies in the use of a quasi-experimental design without a control group, which reduces the ability to establish strong causal relationships based on the results. Thus, the perceived development may be attributed to the mere passage of time rather than to the SCP subject itself. Furthermore, certain individual factors, such as students’ prior experiences or their level of motivation, may have influenced the outcomes.
In addition, the sample was not randomly selected, which may introduce bias due to the intervention being conducted with a very specific sample from a private university in Madrid. There is also some data loss (non-response rate), which was addressed through imputation. However, sensitivity analyses indicated that both the original and imputed samples yielded similar results. Finally, a larger sample size is needed to achieve convergence in mixed models and to explore additional effects.
For future research, it is recommended to employ mixed methods to complement the findings; to triangulate self-perceived development measured by the BGCQ with other assessment methodologies; to implement longitudinal studies to evaluate the persistence of competences over time; and to include control groups to yield more robust and reliable results.

## Data Availability

The data supporting the findings of this study are available on request to the corresponding author.
