# Supplementary material for: Impact of an integral accompaniment program on undergraduate students’ self-perception of transversal competence development: a quasi-experimental study
Source: Front Psychol. 2026 Jan 16;16:1653779. doi: 10.3389/fpsyg.2025.1653779 (PMC12857061; doi:10.3389/fpsyg.2025.1653779)
Supplement: Supplementary file 1 [file Supplementary_file_1.docx]

**Annex 1. BGCQ**

**Annex 2. Differences between pre-test/post-test moments in the different faculties.**

| Faculty | Dimension | **Overall** | **PRE-TEST** ^1^ | **95% CI**^2^ | **POST-TEST**^1^ | **95% CI**^2^ | ***p***^3^ | **Cohen´s *d*** |
| --- | --- | --- | --- | --- | --- | --- | --- | --- |
| Medicine | Intrapersonal | 90.2 ± 8.4 | 89.0 ± 7.9 | 88, 90 | 91.3 ± 8.7 | 90, 92 | **<0.001** | -0.277 |
|  | Interpersonal | 94.3 ± 7.7 | 93.6 ± 7.3 | 93, 95 | 94.9 ± 8.0 | 94, 96 | **0.003** | -0.168 |
| Experimental Sciences | Intrapersonal | 87.1 ± 9.2 | 84.2 ± 8.4 | 83, 85 | 90.0 ± 9.0 | 89, 91 | **<0.001** | -0.666 |
|  | Interpersonal | 90.8 ± 8.9 | 89.0 ± 8.6 | 88, 90 | 92.6 ± 8.9 | 92, 93 | **<0.001** | -0.405 |
| Health Sciences | Intrapersonal | 89.1 ± 8.5 | 87.4 ± 8.0 | 86, 89 | 90.8 ± 8.7 | 89, 92 | **<0.001** | -0.408 |
|  | Interpersonal | 92.0 ± 8.5 | 91.4 ± 8.6 | 90, 93 | 92.7 ± 8.4 | 91, 94 | 0.064 | -0.153 |
| Communication Sciences | Intrapersonal | 87.9 ± 9.7 | 88.2 ± 10.8 | 86, 89 | 87.6 ± 8.5 | 86, 90 | 0.5 | 0.061 |
|  | Interpersonal | 90.5 ± 9.5 | 89.9 ± 10.7 | 89, 93 | 91.0 ± 8.2 | 88, 92 | 0.2 | -0.112 |
| Law, Business and Government | Intrapersonal | 88.7 ± 9.8 | 87.6 ± 8.8 | 86, 89 | 89.8 ± 10.6 | 88, 92 | **0.006** | 0.222 |
|  | Interpersonal | 91.2 ± 8.6 | 91.0 ± 8.1 | 90, 92 | 91.3 ± 9.1 | 90, 93 | 0.7 | 0.036 |
| Advanced Polytechnic School | Intrapersonal | 87.8 ± 9.0 | 87.6 ± 8.5 | 86, 89 | 88.1 ± 9.6 | 86, 90 | 0.5 | 0.054 |
|  | Interpersonal | 89.1 ± 9.9 | 90.4 ± 7.8 | 89, 92 | 87.7 ± 11.5 | 86, 90 | **0.003** | -0.276 |
| Education and Psychology | Intrapersonal | 90.8 ± 8.9 | 90.9 ± 7.3 | 89, 93 | 90.7 ± 10.3 | 88, 94 | 0.9 | -0.021 |
|  | Interpersonal | 93.1 ± 8.9 | 92.8 ± 8.7 | 90, 95 | 93.5 ± 9.3 | 91, 96 | 0.6 | 0.081 |
